# Quantifying the Positional Deviation Between the True Flexion-Extension and Epicondylar Axes of the Elbow: A 3D Computational Study

**DOI:** 10.7759/cureus.70816

**Published:** 2024-10-04

**Authors:** Jonathan Zhi-Wei Gan, Pivatidevi Pareatumbee, Andy Yew, Zehao Tan, Don Thong Siang Koh, Tet-Sen Howe, Suang-Bee Joyce Koh, Bernard Morrey, Yeong Huei Ng

**Affiliations:** 1 Academic Clinical Program-Musculoskeletal Sciences, Singapore General Hospital, Singapore, SGP; 2 Department of Orthopaedic Surgery, Singapore General Hospital, Singapore, SGP; 3 Division of Musculoskeletal Sciences, Singapore General Hospital, Singapore, SGP; 4 Vascular and Interventional Radiology, Singapore General Hospital, Singapore, SGP; 5 Orthopaedic Surgery, Mayo Clinic, Rochester, USA

**Keywords:** 3d modelling, elbow flexion axis, humerus, implant alignment, total elbow replacement

## Abstract

Background and objective

The epicondyles are commonly used surgical reference landmarks for elbow arthroplasty and external fixator application. This study aimed to investigate whether the epicondylar axis differed from the elbow’s true flexion-extension (F-E) axis in terms of both rotational difference and translational offset.

Methods

Three-dimensional (3D) models of 15 cadaver elbows were created. The epicondylar, true F-E, and distal humeral axes were defined using the medial and lateral epicondyles and the normal vector through the trochlear groove’s center respectively. Rotational difference along internal-external, varus-valgus, and flexion-extension rotation plane and translational offset in the anterior-posterior (A-P), medial-lateral (M-L), and inferior-superior (I-F) direction with reference to the distal humerus's long axis were measured.

Results

Minimal rotational differences of 1.9 ± 4.5, 2.1 ± 3.4, and 0.5 ± 2.7 degrees for flexion-extension, varus-valgus, and internal-external rotation were obtained respectively. Considerable translational offsets greater than 10 mm were found for the absolute medial and lateral translational offset with a statistically significant difference recorded in the M-L direction.

Conclusions

Small rotational differences exist between the epicondylar and true F-E axes. Significant differences are observed in the translational offset in the M-L direction and should be considered during implant alignment in order to reduce malalignment and prevent failure.

## Introduction

The medial and lateral epicondyles are commonly employed surface and anatomical landmarks for elbow surgeries, such as the application of elbow external fixators and total elbow arthroplasty (TEA) [1, 2]. The Coonrad-Morrey implant is a widely used, semi-constrained TEA implant, which utilizes these landmarks. An imaginary line joining the medial and lateral epicondyles forms the epicondylar axis and is assumed to approximate the true flexion-extension (F-E) axis of the elbow [1]. However, from an anatomical standpoint, the epicondylar axis should not be assumed to be identical to the true F-E axis, which is instead closely related to the trochlea and capitellum [3].

Deviation from the true F-E axis may alter elbow kinematics and may in turn reduce the efficiency of movement following implantation with an improper alignment subsequently affecting clinical outcomes [4-7]. Several studies have investigated the accuracy of recreating the natural F-E axis [4-7]. Typically, the central axis of the trochlea is used as a reference for the anatomical elbow flexion axis. To identify the flexion axis of the elbow, surgeons have attempted to overlap the capitellum and trochlear contour with their respective centers. However, the anatomical bow of the distal humerus and limb’s position was seen to affect the lateral X-ray projection and resulted in errors when identifying the flexion axis [8].

Various theories of the mechanism of implant failure have been put forward. The failure to align the prosthesis in an anatomically and biomechanically optimal manner may impose higher mechanical stress on implant components, ultimately leading to aseptic loosening, early failure, and chronic pain [9-11]. Malpositioning of an implant may be a crucial underlying factor leading to alterations in joint motion and load distribution with malrotation or malalignment of TEA components being the primary cause of revision in 1-2.3% of TEAs [11-14]. A study conducted in a laboratory setting has illustrated that a malpositioning error of up to 10.2 degrees of internal-external rotation and 9.6 degrees of varus-valgus orientation can occur when determining the flexion axis, thus indicating a risk of error when identifying the landmarks under non-ideal conditions during surgery [11].

Previous studies have mainly focused on the rotational differences between the two axes [7,15]. However, the translational offset of the true F-E axis with respect to the epicondylar axis remains scarcely documented. Therefore, the objective of this study was to quantify and compare both the rotational difference and translational offset between the epicondylar axis and the true F-E axis to determine whether any significant deviation between the measured parameters exists. Subsequent findings may help to improve component positioning, potentially leading to a bone-implant construct that more closely replicates the natural elbow joint kinematics.

## Materials and methods

Establishment of 3D bone models

CT scans of 15 fresh-frozen cadaveric elbow specimens (eight males and seven females; age range: 50-75 years) with no radiological evidence of arthritis, with a scan resolution of 0.6 mm and pixel resolution of 512 × 512, were taken with the elbow in a maximally extended supine position. The scan data were stored in the Digital Imaging and Communications in Medicine (DICOM) format. Three-dimensional (3D) models were created by further processing of the DICOM data through the extraction of the region of interest using an image segmentation software, 3D Slicer (Version 4.6, https://www.slicer.org/), and a threshold between 300 and 1300 Hounsfield unit [16]. Scanning artifacts were visually identified and thereafter manually removed from the region of interest using ImageJ’s eraser tool (ImageJ, National Institutes of Health, Bethesda, MD). Following this, the segmented humerus and ulna were exported to MATLAB 2012b (The MathWorks, Inc, Natick, MA) to obtain the contour of the distal humerus and proximal ulna utilizing thresholding and smoothing techniques available in the Image Processing toolbox.

Creation of epicondylar, distal humeral, and true F-E axis

Epicondylar Axis

**Figure 1:**
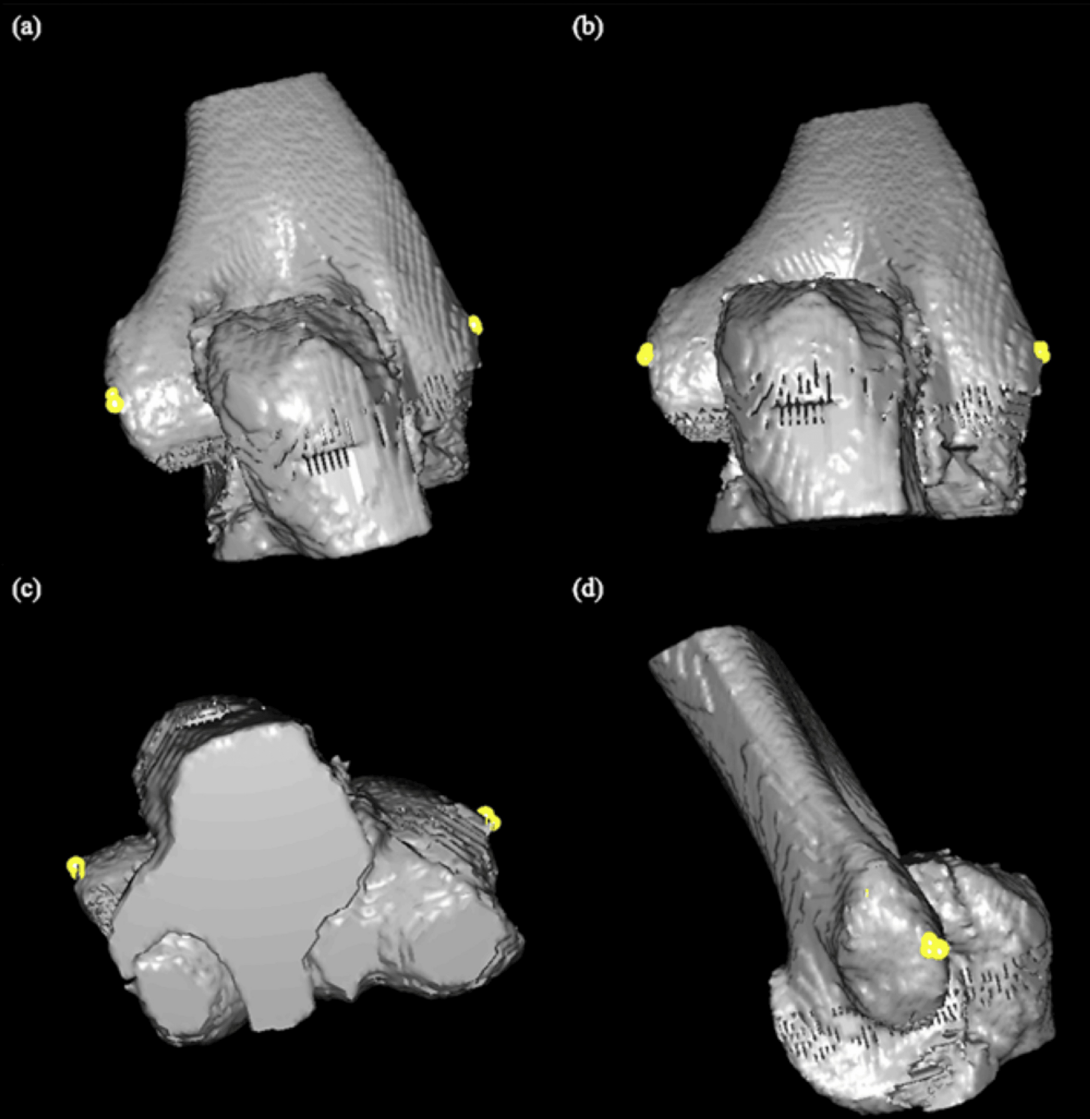
Manual definition of the epicondylar axis (a) Three points were placed on each epicondyle simulating palpable landmarks used by surgeons. (b), (c), and (d) The segmented points in the coronal, axial, and sagittal anatomical planes respectively

The medial and lateral epicondyles, which are common surgical landmarks used to estimate the F-E axis of the elbow [1], were identified on the 3D elbow models. The medial and lateral epicondyles were defined as the apex of the most medial and lateral aspects of the distal humerus respectively, on a plane perpendicular to that of the long axis of the humerus. Three points to approximate the location of each epicondyle were manually chosen by an orthopedic surgeon on the models, consistent with the clinically utilized surgical reference points of the medial and lateral epicondyles, and their averages were calculated. The epicondylar axis was then defined as the axis drawn between the epicondyles. Figure 1 shows an example of the process.

Distal Humeral Axis

The long (intramedullary) axis of the distal humerus was identified as a reference axis. CT slices showing the distal diaphysis and metaphysis were determined manually. From these, five evenly spaced axial slices were selected, and the bony contours of the humerus were obtained. An ellipse was then fitted to these borders, using the least squares method [17] to minimize radial deviation, as follows:

P(t) = α cos(t)u + b sin(t)(n x u) + C

Where t is the radial deviation, a and b are the radii of the ellipse, C is the center of the ellipse, n is the normal vector from the ellipse, and u is the unit vector perpendicular to n.

The long axis of the distal humerus was defined as the line of best fit through the center of the five ellipses, C, using the least squares method and the line equation below:

X = X_0_ + k_ v_

Where X_0_ is a point passing through the line, v is the unit vector of the direction of the line and k is a constant.

True Flexion-Extension Axis of the Elbow

**Figure 2:**
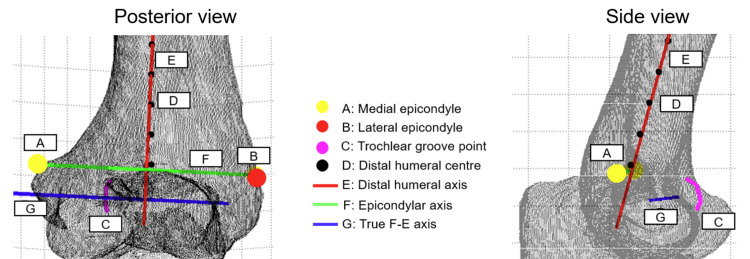
3D elbow diagram with axes plotted a) The medial epicondyle (A) and lateral epicondyle (B) were used to define the epicondylar axis (F). b) The distal humerus axis (E) was defined as the line extending across the distal humeral centers (D). c) The true F-E axis (G) was defined using the trochlear groove points (C) and its normal vector

We defined the true F-E axis of the elbow as a normal vector through the center of the trochlear groove [3,18]. The model was first positioned in the posterior view and then rotated from the posterior to the side view with the medial epicondyle facing the viewer. To define the trochlear groove, a starting point and an endpoint were chosen according to the curvature of the trochlear groove. Twenty points on the deepest part of the trochlear groove were identified based on standard anatomical landmarks. These points were fitted to a circle using the parametric equation of a circle derived from the equation of the ellipse above with a = b. The errors associated with the circle fitting range between -0.21 and 0.62 for all specimens. The Gauss-Newton algorithm was used to minimize the sum of squared radial deviations [7]. Using this method, the center of the circle, C, and its normal were obtained. The true F-E axis was taken to be the normal vector of the circle, n. A visual representation of how the various axes were derived from the 3D elbow model is presented in Figure 2.

Comparison between F-E and epicondylar axis

To compare the true F-E axis and epicondylar axis, the rotational difference refers to the difference in orientation of the rotational axes with respect to the X, Y, and Z planes and the translational offset defined as the movement of a point and axis in a given direction were measured.

Definition of Anatomical Planes

All axes were projected onto the anatomical planes of the elbow using the dot product and cross product to obtain the rotational difference and translational offset in all directions. The coronal plane was defined as being parallel to both the long axis and the F-E axis. The sagittal plane was defined as being perpendicular to the coronal plane and the F-E axis. The axial plane was described as a plane transecting the long axis of the distal humerus.

Quantifying the Rotational Difference

**Figure 3:**
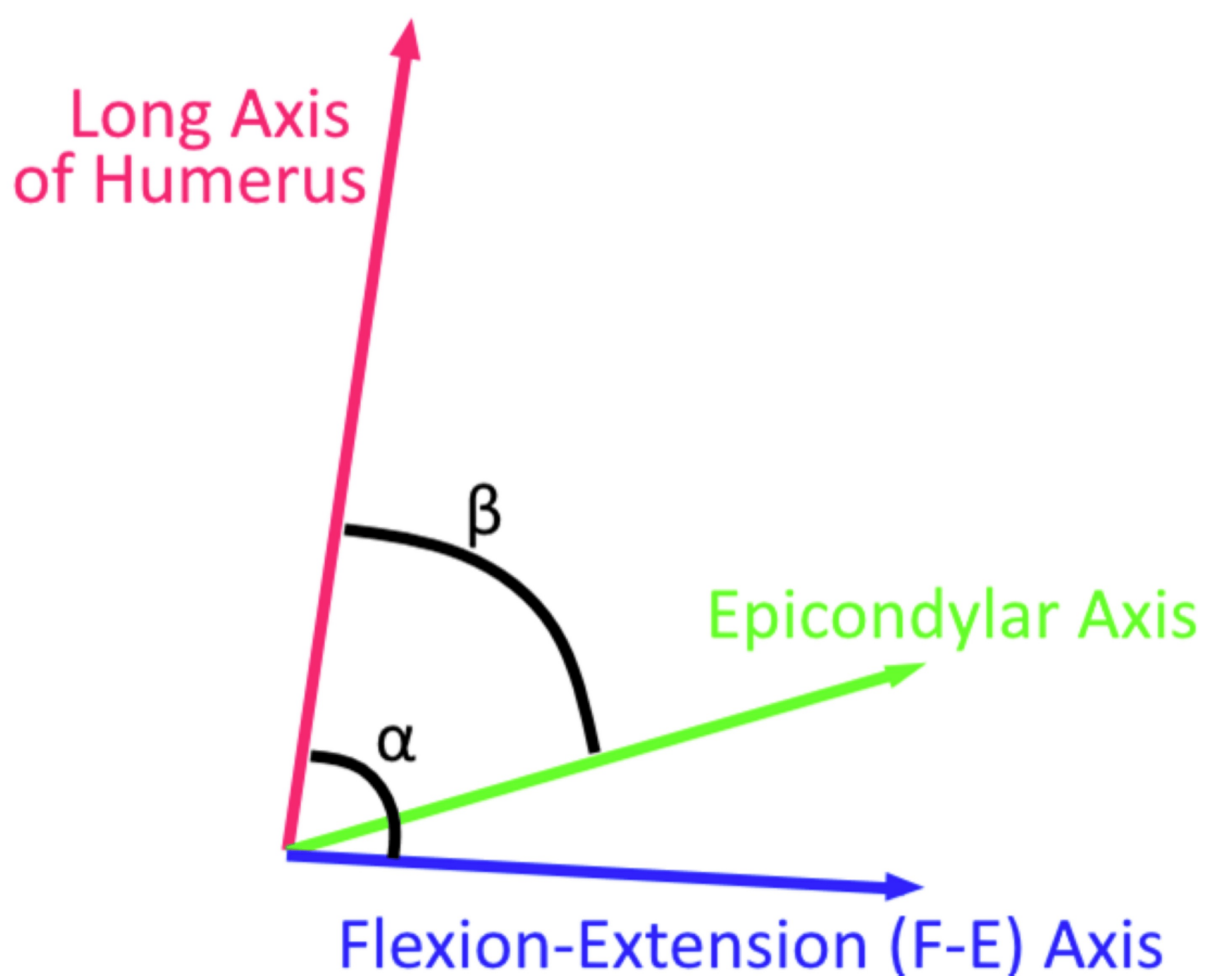
Comparison of axes. Angle between epicondylar axis and F-E axis: |ɑ - ß| ɑ: Angle between the long axis of the humerus and the true flexion-extension axis. ß: Angle between the long axis of the humerus and epicondylar axis; angular difference calculated as ɑ - ß

The angles between the long axis and the true F-E axis and the angle between the long axis and epicondylar axis were obtained and named ɑ and ß respectively. The orientation of ɑ and ß were compared to previously reported angles in existing studies [19]. The angle between the true F-E axis and the epicondylar axis was calculated as ɑ - ß (Figure 3). Rotational differences in the coronal plane are termed internal/external rotation; in the sagittal plane, they are referred to as flexion/extension, and in the axial plane, they are denoted as varus/valgus. The rotational measurements were then repeated in each plane.

Quantifying Translational Point Offset

**Figure 4:**
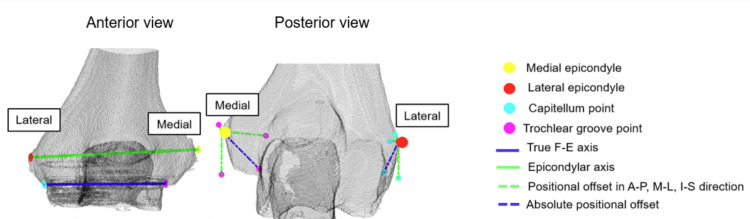
Projection of the trochlear and capitellum point onto anatomical planes to measure the translational offset between the projected points and the respective epicondyles a) Relative position of the epicondylar axis and true F-E axis in the anterior view. b) Positional offset between the trochlear and capitellum points relative to that of the medial and lateral epicondyle in the absolute, anterior-posterior (AP), medial-lateral (ML), and inferior-superior (IS) directions in the posterior view

**Figure 5:**
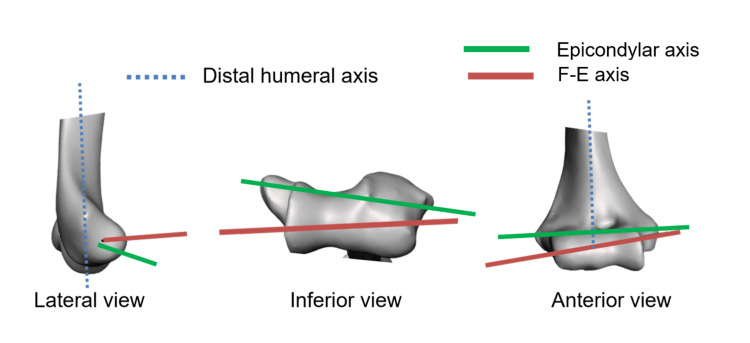
Projection of flexion-extension axis in anatomical views Illustration of the distal humeral axis and true flexion-extension (F-E) axis in the lateral, inferior, and anterior views

The points where the true F-E axis intersected/exited the humerus at its medial and lateral boundaries were named the trochlear and capitellum points respectively. The absolute translational offset between these points and the nearest epicondyle was calculated, as well as with respect to the three anatomical planes, with each plane providing two possible directions of translation namely anterior-posterior (A-P), medial-lateral (M-L), and inferior-superior (I-S). Figure 4 shows the relative position of the two axes, and the position of the trochlear and capitellum points relative to that of the medial and lateral epicondyle respectively. The medial translational offset was defined as the offset of the trochlear point from the medial epicondyle and the lateral translational offset was defined as the offset of the capitellum point and from the lateral epicondyle. Figure 5 demonstrates the projection of the F-E axis under the different anatomical views.

Quantifying Axis Translational Offset

The overall axis offset between the epicondylar and true F-E axis was studied by translating the true F-E axis from its original location, such that it extended through the lateral epicondyle instead of the capitellum point. The point where the translated true F-E axis intersected the medial humerus was recorded and known as the medial humerus intersection point. The absolute translational offset between the medial humerus intersection point and the nearest epicondyle, as well as with respect to the three anatomical planes were calculated.

Statistical analysis

To assess the intra-observer and inter-observer reliability, six parameters comprising X, Y, and Z coordinates for the medial and lateral epicondylar anatomical landmarks were obtained. The parameters were measured by three co-authors in six humeral specimens on five different days. The intra-class correlation coefficients were computed using R (Version 4.3.3) and the “Psych” package. Data were checked for normality using the Shapiro-Wilk test before statistical analysis was performed. The p-values of all data were more than 0.05 (range: 0.051 to 0.959), indicating a normal distribution. Rotational comparisons and comparisons of the translational offset between points were then performed using the student’s t-test (α = 0.05). Statistical analysis was performed using SPSS Version 24 (IBM Corp., Armonk, NY; released 2016). The study was conducted between July 22, 2011, and July 21, 2012, at Singapore General Hospital in Singapore.

## Results

Intra-observer and inter-observer ICC

There was excellent absolute agreement (ICC2 = 1, p<0.001) using the two-way mixed effect models and “single random rater” unit when assessing the inter-observer ICC for both medial and lateral epicondylar anatomical landmarks. When comparing for intra-observer ICC, excellent absolute agreement (rater 1: ICC3 = 1, p<0.001; rater 2: ICC3 = 1, p<0.001; rater 3: ICC3 = 1, p<0.001) using the two-way mixed effect models and “single fixed rater” unit were computed for each rater for both medial and lateral epicondylar anatomical landmarks.

The rotational deviation between the epicondylar axis and the true flexion-extension (F-E) axis

The mean and standard deviation (SD) for the raw angle between the epicondylar and true F-E axis with respect to the long distal humeral axis and in our study were 85.9° ± 5.3° and 83.7 ± 3.8°. It can be observed that the measurement of the rotational difference within the three rotation planes of the elbow yielded similar results, with no statistically significant difference found between the epicondylar angle and F-E angle (Table 1).

**Table 1:** Rotational differences between the epicondylar axis and true F-E axis Table presenting the mean and standard deviation (SD) of the angular/rotational differences being studied. Columns are divided into the absolute angle and the three different anatomical planes namely flexion/extension, varus/valgus, and internal/external rotation. P-values were calculated using the Student’s t-test

	Absolute angle		Flexion (+)/extension (-)		Varus (+)/valgus (-)		External (+)/internal (-)	
	Mean	SD	Mean	SD	Mean	SD	Mean	SD
Epicondylar angle (ß)	85.9	5.3	86	8.8	92.5	7.1	83.2	6.4
F-E angle (ɑ)	83.7	3.8	84.1	7.2	90.5	7.2	82.7	6.4
Difference (|ɑ - ß|)	2.2	4.8	1.9	4.5	2.1	3.4	0.5	2.7
P-value	0.204		0.523		0.442		0.851	

Point translational offset

The absolute medial translational offset was 23 ± 3.4 mm while the lateral translational offset was 16 ± 3.6 mm (p<0.001). Based on the results, a relatively similar translational offset was observed for both the medial and lateral translational offsets in the A-P and I-S directions. However, in the M-L direction, the medial translational offset with a magnitude of -12.3 ± 3.4 mm was notably larger than the lateral translational offset with a magnitude of 3.1 ± 1.6 mm with statistically significant differences only recorded in the absolute and M-L translational offset (p<0.001). Table 2 illustrates the average projected translational offset of the trochlear and capitellum points from the respective epicondyles along the three directions.

**Table 2:** Absolute and projected translational offset between the epicondyles and the respective trochlear and capitellum points Table presenting the mean and standard deviation (SD) of the positional/translational offsets being studied between the epicondyles and the respective trochlear and capitellum points. Columns are divided into the absolute translational offset and the three different anatomical directions namely anterior/posterior, medial/lateral, and distal/proximal. P-values were calculated using the Student’s t-test

	Absolute		Anterior (+)/posterior (-)		Medial (+)/lateral (-)		Distal (+)/proximal (-)	
	Mean	SD	Mean	SD	Mean	SD	Mean	SD
Medial positional offset (mm)	23	3.4	14.1	4	-12.3	3.4	12.1	5.3
Lateral positional offset (mm)	16	3.6	11.4	4.1	3.1	1.6	9.2	5.2
P-value	<0.001		0.068		<0.001		0.138	

Axis translational offset

The absolute translational offset of the F-E axis intersection with the medial epicondyle was 11.1 ± 7.3 mm. The average translational offset was 8.8 ± 7.4 mm in the A-P direction, 1.4 ± 1.7 mm in the M-L direction, and 9.4 ± 6.1 mm in the I-S direction. Similar translations greater than 8 mm were observed in the A-P and I-S direction whereas only a small translation was observed in the M-L direction.

## Discussion

To establish our models’ accuracy, we compared the overall F-E angle and rotational difference against the values in the existing literature. Our calculated F-E angle demonstrated a difference of 4.1% compared to the measurement reported by Brownhill et al. [19]. In addition, the absence of significant statistical difference in the absolute angle as well as the rotational difference when compared to values published by Brownhill et al. [19] (p=0.327) and Sabo et al. [7] (p=0.479) respectively indicates that the developed model agreed with the values in the literature and is thus considered valid.

For the translational offset, our results indicated that the trochlear and capitellar points were offset from their respective epicondyles with all three directions exhibiting considerable deviations close to 10 mm. A noticeable difference was observed in the M-L direction with the medial translational offset being four times the lateral translational offset with a statistically significant difference only recorded in the M-L direction. Furthermore, an overall axis translational offset of 11.1 mm existed between the true FE axis and the ML axis. Previous research has illustrated that malpositioning of the hinge by 10 mm can result in 10 times the level of motion resistance [20]. Therefore, our findings suggest that increased mechanical stress can be imposed on the bearing surface due to suboptimal implant alignment. This may result in particulate debris generation and eventually aseptic loosening [21,22], which remained the most common reason for revision as reported across multiple registries [14,19,23-26].

For the rotational differences, our findings illustrated minimal differences ranging from 0 to 3 degrees in all planes with the F-E and V-V planes experiencing four times the rotational difference compared to the E-I plane with no statistically significant differences recorded. Nevertheless, it is important to note that improper rotational alignment is known to lead to joint incongruity, constant maltracking, or further malrotations, which may be magnified after repeated wear cycles of flexion and extension [6]. A study of non-constrained implants (Kudo) showed that valgus inclination resulted in polyethylene wear and early failure [27]. Besides, humeral component malrotation exceeding 10 degrees of internal or external rotation has also been shown to be sufficient to reach the valgus or varus design limit of a semi-constrained implant (Coonrad-Morrey) and can lead to early failure [28]. Hence, the minor rotational differences observed cannot be ignored; otherwise, it may consequently lead to malalignment and, eventually, early implant failure.

Accounting for these rotational and translational differences is vital to reduce stress on the implant and prevent early implant failure, even in semi-constrained implants which may alter the normal elbow kinematics and have a negative impact on the overall loading in the bone-implant construct. Additionally, patient non-compliance with loading restrictions [29] can also further amplify any deleterious effects. However, the task of discerning the true F-E axis during surgical procedures also undoubtedly presents a formidable challenge, primarily due to the geometrical complexity characterizing the elbow joint as well as varying anatomical profiles across different patient groups. The true F-E axis has been previously determined to be a line through the center of the trochlea and capitellum by Morrey and Chao, but reliably identifying these points remains a challenge, even for experienced surgeons [3].

The medial and lateral epicondyles, as well as the flat of the posterior condylar surfaces, are the frequent anatomical landmarks used for purposes of total replacement and applications of elbow dynamic external fixators [3]. The use of these surrogate landmarks is necessary due to varying reasons. The medial and lateral epicondyles are easily palpable and referenced landmarks during surgery. Moreover, traumatic injuries to the elbow such as distal humeral fractures may sometimes lead to the loss of anatomical landmarks at the articular surface.

Alternative landmarks have been proposed, which have their own limitations with the recommended landmarks dependent on the surgical guide. In a more recent design, the Zimmer Nexel TEA [30] recommends yet another set of landmarks, using a point at the anterior/inferior aspect of the medial condyle, and the center of the capitellum laterally. Sabo et al. [7] found the flat portion of the posterior humeral cortex that was directly proximal to the olecranon fossa to be a reproducible landmark for rotation. However, this landmark varied significantly between genders and was found to be difficult to identify by surgeons.

The difficulty in practically measuring the true F-E axis during an actual surgical procedure cannot be overlooked. Future advancements in implant design, surgical techniques, or jig design that allow better alignment to the true F-E axis of the elbow can potentially lead to improved longevity of total elbow prosthesis to maximize alignment with the natural axis of the elbow, thereby optimizing overall joint movement. Thus, implementing this concept into clinical practice will necessitate the development of surgical jigs capable of predicting the intended landmarks or preoperative surgical planning to acquire 3D bone models, with automated algorithms computing the implant alignment according to the true F-E axis with the aid of a computer navigation system to perform implantation.

There are several advantages of using 3D computer modeling to virtually investigate both the rotational and translational differences between epicondylar and the true F-E axis. Firstly, this is a cost-effective method as well as a more accurate means of obtaining the data as opposed to physical experimental testing. Additionally, measuring the desired parameters using actual cadavers may prove to be even more challenging with a greater risk of inaccuracy as conventional measuring instruments may not be able to measure non-geometric shapes and contours.

This study has a few limitations. One limitation is the relatively small sample size of 15 from cadavers of only Caucasian origin, coupled with a limited age range. Hence, samples are not representative of the full spectrum of anatomical variation. Further research involving a larger number of samples from various ethnicities and age groups should be undertaken. Another limitation is the lack of suitable validation of the results obtained owing to the scarcity of relevant data in the literature not allowing sufficient comparisons to be made. Moreover, a sensitivity analysis was not conducted to determine the effect of rotational orientation on the position of the F-E axis. Nonetheless, the reliability of the translational offset will require further study. If it is consistent regardless of individual demographics, the medial and lateral epicondyles may be used as references, with an offset applied to achieve accurate alignment.

Further exploration of bone-implant mechanics requires additional research to determine the extent to which prosthesis misalignment affects mechanical stresses and strains during activities of daily living, by using finite element analysis or physical experimental testing. If there are significant differences in the bone-implant interaction due to the deviation between the epicondylar and true F-E axis, surgeons may consider aligning implants to the true F-E axis instead of the epicondylar axis. To aid and enhance the alignment precision, implant designers can incorporate an offset correction into new surgical jig designs. Furthermore, the development of implants that closely emulate the joint's natural kinematics may yield improved patient-reported outcomes and clinical results.

## Conclusions

There are differences in both rotational and translational values between the true F-E axis and the epicondylar axis, with the most significant translational offset recorded in the mediolateral direction. Therefore, to avoid potential malalignment, the true F-E axis must be used as the reference axis, instead of the epicondylar axis.
